# Protein Kinase D1 Maintains the Epithelial Phenotype by Inducing a DNA-Bound, Inactive SNAI1 Transcriptional Repressor Complex

**DOI:** 10.1371/journal.pone.0030459

**Published:** 2012-01-20

**Authors:** Ligia I. Bastea, Heike Döppler, Bolanle Balogun, Peter Storz

**Affiliations:** Department of Cancer Biology, Mayo Clinic, Jacksonville, Florida, United States of America; National Cancer Center, Japan

## Abstract

**Background:**

Protein Kinase D1 is downregulated in its expression in invasive ductal carcinoma of the breast and in invasive breast cancer cells, but its functions in normal breast epithelial cells is largely unknown. The epithelial phenotype is maintained by cell-cell junctions formed by E-cadherin. In cancer cells loss of E-cadherin expression contributes to an invasive phenotype. This can be mediated by SNAI1, a transcriptional repressor for E-cadherin that contributes to epithelial-to-mesenchymal transition (EMT).

**Methodology/Principal Findings:**

Here we show that PKD1 in normal murine mammary gland (NMuMG) epithelial cells is constitutively-active in its basal state and prevents a transition to a mesenchymal phenotype. Investigation of the involved mechanism suggested that PKD1 regulates the expression of E-cadherin at the promoter level through direct phosphorylation of the transcriptional repressor SNAI1. PKD1-mediated phosphorylation of SNAI1 occurs in the nucleus and generates a nuclear, inactive DNA/SNAI1 complex that shows decreased interaction with its co-repressor Ajuba. Analysis of human tissue samples with a newly-generated phosphospecific antibody for PKD1-phosphorylated SNAI1 showed that regulation of SNAI1 through PKD1 occurs *in vivo* in normal breast ductal tissue and is decreased or lost in invasive ductal carcinoma.

**Conclusions/Significance:**

Our data describe a mechanism of how PKD1 maintains the breast epithelial phenotype. Moreover, they suggest, that the analysis of breast tissue for PKD-mediated phosphorylation of SNAI1 using our novel phosphoS11-SNAI1-specific antibody may allow predicting the invasive potential of breast cancer cells.

## Introduction

E-cadherin mediated cell-to-cell contacts are important to the structural integrity of epithelial cell layers. During tumorigenesis epithelial tumor cells lose E-cadherin expression concomitantly with acquisition of mesenchymal characteristics. This process has been termed epithelial-mesenchymal transition or EMT [1]. Cells undergoing EMT acquire a fibroblast-like shape and show increased motility and invasiveness due to activation of a mesenchymal gene expression program [2]. EMT-associated cellular changes are loss of adherens junctions and epithelial cell polarity, cell scattering due to loss of cytokeratin expression, increased expression of N-cadherin and αvβ6 integrin, and increased secretion of fibronectin and matrix metalloproteinases [3], [4].

In many types of cancers and tumor cell lines, transcriptional repression by SNAI1 family members has emerged as a key mechanism for the dynamic modulation of E-cadherin expression and the induction of EMT [1], [5], [6], [7]. Other SNAI1 target genes encoding proteins maintaining the polarized epithelial structure include cytokeratins 17/18 [8], collagen 2α1 [9], Mucin1 (MUC1), ZEB1 [8] and SNAI1 itself [10]. Several studies have correlated SNAI1 expression with tumor growth and invasion [11], lymph node metastasis [12], [13], effusion [14], [15], distant metastasis [16], [17], [18], chemoresistance [19] and the recurrence of tumors [20].

The SNAI1 family of transcriptional repressors consists of three members SNAI1 (Snail1, Snail), SNAI2 (Slug, Snail2) and SNAI3 (Smuc, Snail3). They all share a common structural organization consisting of a highly conserved C-terminal region, which harbors four zinc fingers functioning as sequence specific DNA-binding domains for E2-box type DNA sequences C/A (CAGGTG) [1], [4]. Dependent on the target gene, repressor capacity requires SNAI1 interaction with the co-repressors Ajuba, PRMT5 and SIN3a, or histone deacetylases 1 and 2 (HDAC) [21], [22], [23]. Some of these interactions (i.e. binding to Ajuba) are dependent on the SNAG-domain at the N-term [23], [24]. SNAI1 can be regulated at the transcriptional level, but also at the protein level. For example, the kinase GSK3β phosphorylates SNAI1 at several serine residues in its NES (nuclear export sequence) and its destruction box leading to SNAI1 nuclear export, ubiquitination and degradation [7], [18], [25], [26]. However, in many cells GSK3β is negatively-regulated by Akt and thus it is unclear of how SNAI1 is kept in check. Recently, an additional regulation of SNAI1 through phosphorylation at serine 11 was suggested [27], [28], [29]. Phosphorylation of SNAI1 at this site can mediate its nuclear export via binding to 14-3-3σ [27].

Protein Kinase D (PKD) is a serine/threonine kinase that belongs to the family of calcium/calmodulin-dependent kinases (CaM-Ks) [30], [31]. PKD is a sensor for oxidative stress [32], [33], but is also activated by growth factors and subunits of trimeric G-proteins [34] and small RhoGTPases such as RhoA [35], [36]. In invasive ductal carcinoma of the breast PKD1 is downregulated in its expression [37]. Moreover, in breast, prostate and gastric cancer cell lines PKD1 expression and activity reversely correlate with the migratory potential and invasiveness [37], [38], [39]. Several PKD1 targets can contribute to its negative-regulatory function on cell motility. These include the phosphatase slingshot [40], [41], the Par-1 polarity kinase [42], β-catenin and E-cadherin [38], [43].

It is not known if PKD has a role in maintaining the epithelial phenotype of normal cells. Here we show that inactivation of PKD1 in normal mammary gland epithelial cells induces EMT leading to a mesenchymal phenotype. We show that PKD1 maintains E-cadherin levels in epithelial cells by regulating SNAI1 through direct phosphorylation. This leads to a loss of SNAI1 transcriptional repressor activity, although phosphorylated SNAI1 stays bound to its target sequence at the E-cadherin promoter. Moreover, such phosphorylation-mediated regulation of SNAI1 seems to occur *in vivo* in normal breast ductal tissue and is decreased or lost in invasive ductal carcinoma, indicating that the previously reported loss of PKD1 expression in invasive breast cancer may directly transfer to E-cadherin inhibition through SNAI1.

## Results

### PKD1 conserves the epithelial phenotype in normal epithelial mammary gland cells

**Figure 1:**
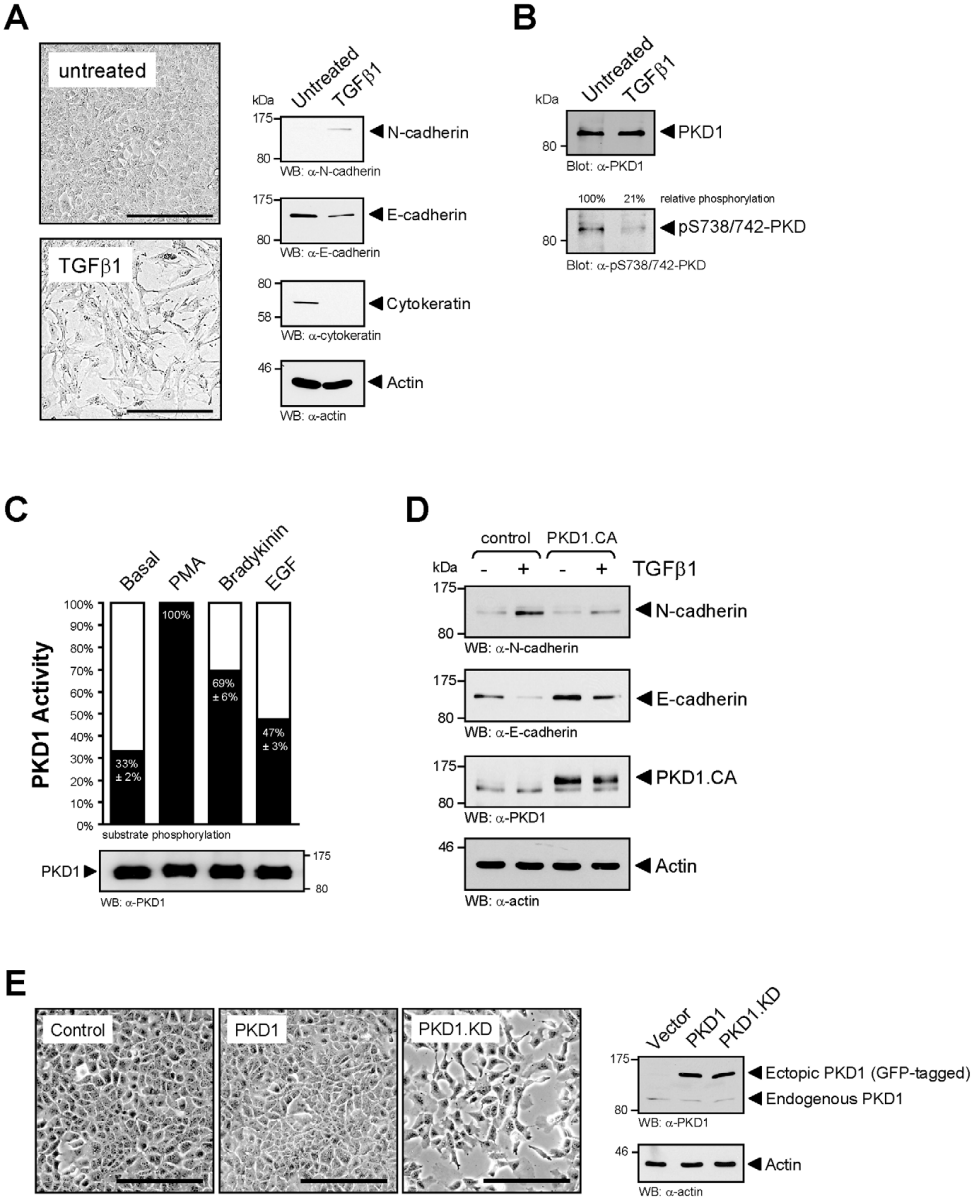
PKD1 conserves the epithelial phenotype in normal mammary gland cells. **A:** NMuMG cells were either left untreated or were treated with TGFβ1 (10 ng/ml) for 48 hours. Cell morphology was photographed (bar is 200 µm) and cells were harvested and analyzed for expression of epithelial (E-cadherin, cytokeratin) and mesenchymal (N-cadherin) markers by Western blotting with anti-N-cadherin, anti-E-cadherin, or anti-cytokeratin antibodies. Staining for actin (anti-actin) served as a loading control. **B:** NMuMG cells were treated with TGFβ1 (10 ng/ml) for 24 hours. Endogenous PKD1 was immunoprecipitated (anti-PKD1) and analyzed for phosphorylation at its activation loop that correlates with its activity (anti-pS738/742-PKD), or samples were control stained for total PKD1 (anti-PKD1). **C:** Cells were stimulated with PMA (100 nM, 10 min), EGF (50 ng/ml, 10 min), Bradykinin (0.5 µg/ml, 10 min) or left untreated. Endogenous PKD1 was immunoprecipitated and subjected to an *in vitro* kinase assay using PKD substrate peptide. PKD1 activity is depicted relative to PMA-activated PKD1 (maximum activation). Equal immunoprecipitation was controlled by SDS-PAGE and immunoblot (anti-PKD1). **D:** NMuMG cells were either transfected with control vector or with active PKD1 (PKD1.CA, PKD1.S738E.S742E). 24 hours after transfection, cells were treated with TGFβ1 (10 ng/ml) for 24 hours. Lysates were analyzed for expression of N-cadherin, E-cadherin, expression of PKD1, or actin as a loading control. **E:** NMuMG cells were stably-transfected with vector control, wildtype PKD1 or kinase-dead PKD1.K612W (PKD1.KD) Cell morphology was analyzed by brightfield microscopy (bar is 200 µm). Expression of endogenous and overexpressed PKD1 was determined by Western blot analysis using an anti-PKD1 antibody. Immunoblotting for actin (anti-actin) served as loading control.

In breast cancer cells PKD1 is a negative-regulator of cell migration and invasion [41]. Consequently, PKD1 is downregulated in its expression in human invasive ductal carcinoma of the breast [37]. Here we investigated if PKD1 can preserve an epithelial phenotype by blocking epithelial to mesenchymal transition of normal breast cells, an initial step that leads to increased cell motility. To test this we used normal murine mammary gland epithelial cells (NMuMG), which in response to TGFβ1 undergo a full EMT, in accordance with morphological changes to a mesenchymal phenotype including upregulation of N-cadherin and downregulation of E-cadherin and cytokeratin expression at the molecular level (**Fig. 1A**). Under normal growth conditions, NMuMG cells show high levels of endogenous, basally-active PKD1 as measured with a phospho-specific antibody (pS738/742-PKD) directed against the phosphorylated PKD activation loop. Treatment with TGFβ1 decreased PKD activity to approximately 20% of its basal activity, suggesting that PKD1 inhibition is one mechanism of how TGFβ1 induces EMT (**Fig. 1B**). By comparing this basal activity to PKD1 activity in cells either stimulated with the phorbol ester PMA (maximum activation) or the PKD1 activators bradykinin and EGF, we found that under normal growth conditions in NMuMG cells approximately one third of the PKD1 pool is in its active form (**Fig. 1C**). Moreover, expression of a constitutively-active PKD1 allele (PKD1.CA, PKD1.S738E.S742E mutant) increased basal E-cadherin levels. It also decreased TGFβ1-mediated EMT as measured by decreased expression of N-cadherin, and maintained basal E-cadherin levels (**Fig. 1D**). When expressing a kinase-dead variant of PKD1 (PKD1.KD, PKD1.K612W mutant) we further found that the inhibition of PKD1 in these cells alone is sufficient to induce an EMT-like cellular phenotype (**Fig. 1E**). These data indicate that PKD1 in normal murine mammary gland cells is constitutively-active in its basal state and prevents EMT.

### PKD1 regulates E-cadherin expression in epithelial cells

**Figure 2:**
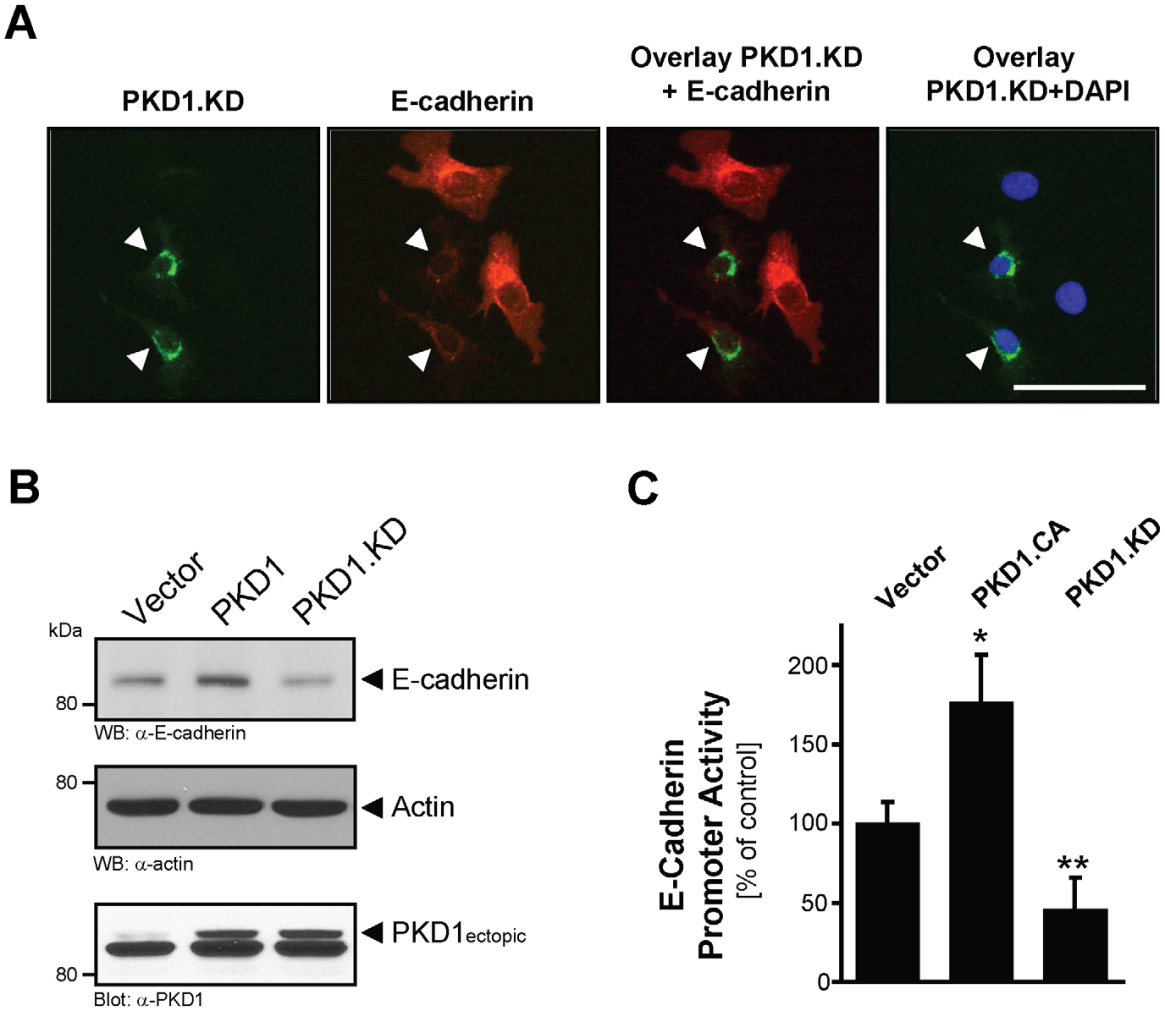
PKD regulates E-cadherin expression in epithelial cells. **A:** NMuMG cells were transfected with GFP-tagged, kinase-dead PKD1 (PKD1.KD) and endogenous expression of E-cadherin was determined with immunofluorescence staining (anti-E-cadherin). DAPI staining served as a nuclear marker (bar is 50 µm). **B:** MCF-7 cells were transfected with vector control, HA-tagged PKD1 or kinase-dead PKD1 (PKD1.KD). After 48 hours, samples were analyzed by Western blot for E-cadherin expression (anti-E-cadherin) as well as expression of PKD1 (anti-PKD1). Staining for actin (anti-actin) served as loading control. **C:** MCF-7 cells were transfected with vector control, HA-tagged constitutively-active PKD1 (PKD1.CA) or kinase-dead PKD1 (PKD1.KD) as well as E-cadherin promoter luciferase gene reporter and renilla luciferase reporter. Induced luciferase activity was measured. Error bars shown represent standard deviations. The asterisks indicate statistical significance (p<0.05) as compared to vector control.

EMT is linked to the loss of expression of E-cadherin, which is a marker for the normal epithelial phenotype. The expression of a kinase-dead PKD1 allele in NMuMG cells correlated with decreased E-cadherin expression (**Fig. 2A**, arrows). Moreover, in other epithelial cell lines including MCF-7 and MDCK, E-cadherin expression was increased when a constitutively-active PKD1 was expressed and decreased when the kinase-inactive PKD1 mutant was expressed (**Fig. 2B** and **Fig. S1**). Finally, the use of an E-cadherin promoter gene reporter demonstrated that PKD1 similarly affects E-cadherin at the gene expression level (**Fig. 2C**).

### Active PKD1 directly phosphorylates SNAI1

**Figure 3:**
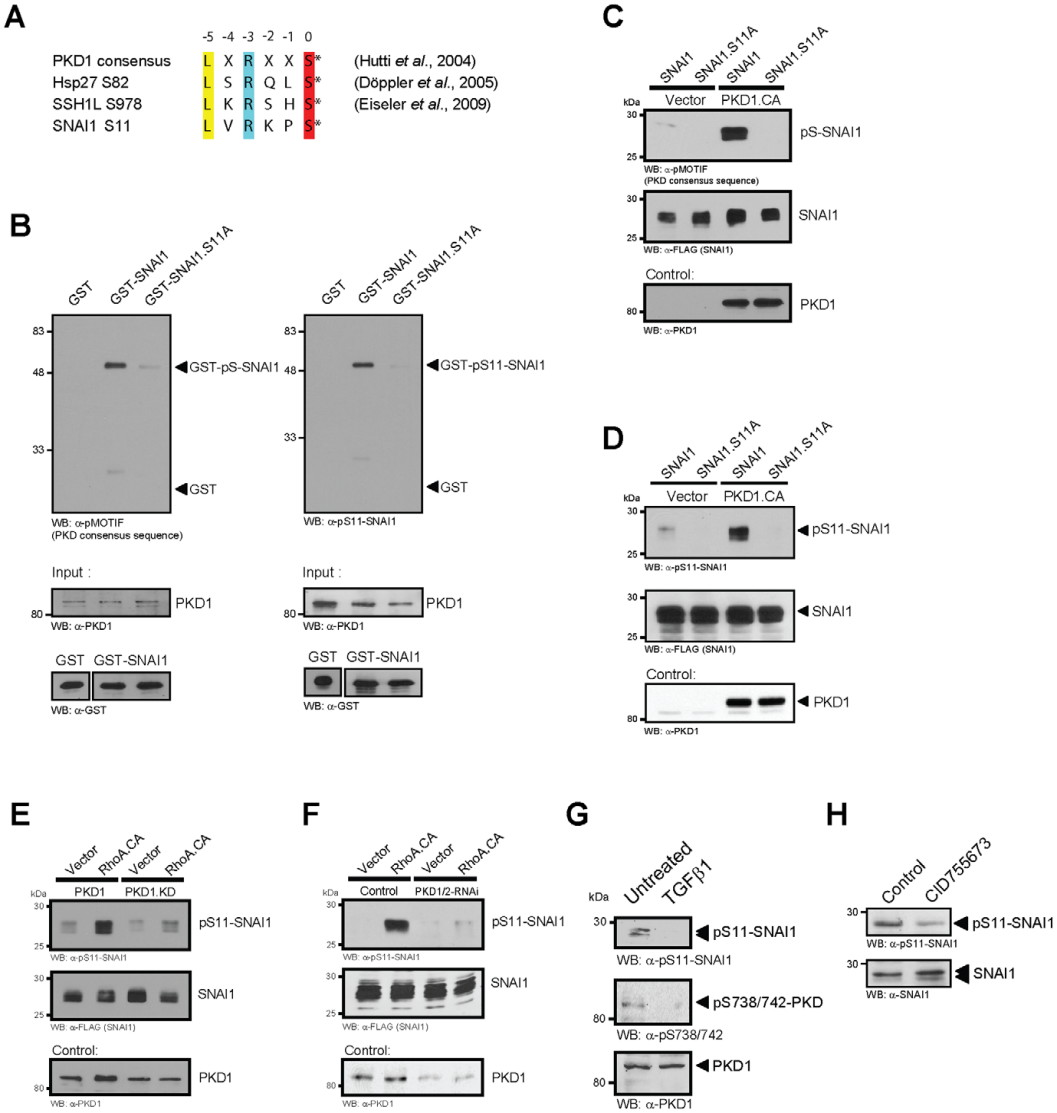
Active PKD1 directly phosphorylates SNAI1 at S11. **A:** The amino-acids surrounding serine 11 in SNAI1 form a PKD consensus motif as it was described for S82 of Hsp27 and S978 of SSH1L. **B:** PKD phosphorylates SNAI1 at S11 in an *in vitro* assay. Bacterially-expressed and purified GST (negative control), GST-SNAI1 or GST-SNAI1.S11A were incubated in a kinase reaction with purified active PKD1. Substrate phosphorylation was detected using the pMOTIF antibody, which recognizes the phosphorylated PKD motif in PKD substrates [45] or with the novel pS11-SNAI1 antibody specifically generated for this site. Control blots were performed for protein input (anti-PKD1, anti-GST). **C, D:** HeLa cells were transfected with combinations of vector control, active PKD1 (PKD1.CA) and SNAI1 or SNAI1.S11A mutant as indicated. PKD-mediated phosphorylation of SNAI1 was detected using the pMOTIF (C) or the pS11-SNAI1 (D) antibodies. **E, F:** HeLa cells were transfected with combinations of vector control, active RhoA (RhoA.CA) and PKD1 or PKD1.KD mutant (E) or control shRNA and shRNA specific for PKD1/2 (F) as indicated and FLAG-tagged SNAI1. PKD-mediated phosphorylation of SNAI1 was detected using the pS11-SNAI1 antibody. Samples were also control-stained for SNAI1 and PKD1 expression using anti-FLAG or anti-PKD1 antibodies, respectively. Anti-GST control staining for RhoA.CA and GST control are depicted in **Figure S2**. **G:** NMuMG cells were treated with TGFβ1 (10 ng/ml) for 48 hours. Total cell lysates were analyzed for phosphorylation of endogenous SNAI1 at S11 (anti-pS11-SNAI1) or PKD1 activity (anti-pS738/742-PKD) or total PKD1 expression (anti-PKD1) as indicated. **H:** NMuMG cells were treated with CID755673 (25 µM, 4 hr) or left untreated as indicated. Total cell lysates were analyzed for phosphorylation of endogenous SNAI1 at S11 (anti-pS11-SNAI1) or SNAI1 expression (anti-SNAI1).

In order to determine if PKD1 maintains the epithelial phenotype through direct phosphorylation of transcriptional repressors of the E-Cadherin gene, we analyzed the sequences of SNAI1, Slug/SNAI2, SNAI3 and Twist for the PKD phosphorylation consensus motif LXRXXS (with X as any amino-acid and S as the targeted serine residue) [44]. Ideal PKD phosphorylation motifs were detected in SNAI1. Of these S11 and its surrounding motif is 100% identical in human, mouse and rat SNAI1 (**Fig. 3A**). In order to demonstrate direct phosphorylation by PKD1, we performed *in vitro* kinase assays using bacterially-expressed, purified wildtype GST-SNAI1 or GST-SNAI1.S11A fusion proteins and baculovirally-expressed and purified PKD1. Using a pMOTIF antibody that recognizes PKD substrates (previously described in [45]), we found that S11 is the only phosphorylation site for PKD1 in SNAI1 (**Fig. 3B** left side). We also generated a phosphospecific antibody for S11-phosphorylated SNAI1 to directly demonstrate phosphorylation by PKD1 (**Fig. 3B**, right side). Moreover, PKD1-mediated phosphorylation of SNAI1 at this residue could be demonstrated in cells with the pMOTIF (**Fig. 3C**) and the pS11-SNAI1 antibody (**Fig. 3D**). To determine the relative stoichiometry of SNAI1 phosphorylation by PKD1 we performed an *in vitro* kinase assay in presence of [γ-^32^P]ATP. PKD1 phosphorylated SNAI1 to ≈1.08±0.05 mol phosphate/mol SNAI1 protein.

We and others previously have shown that PKD1-mediated inhibition of cell motility is mediated through its activation by the RhoGTPase RhoA [35], [36]. Expression of active RhoA increased SNAI1 phosphorylation at S11 and this was blocked with both, kinase-dead PKD1 (**Fig. 3E** **, Figure S2**) as well as when PKD1/2 was knocked-down (**Fig. 3F** **, Figure S2**). Moreover, in NMuMG cells treated with TGFβ1 nuclear PKD1 was inactive (as measured by phosphorylation of its activation loop serines) and this correlated with decreased phosphorylation of SNAI1 at S11 (**Fig. 3G**). Moreover, treatment of NMuMG cells with the PKD inhibitor CID755673 decreased basal SNAI1 phosphorylation at S11 (**Fig. 3H**), further supporting the principal conclusion that active PKD1 may prevent EMT in these cells via phosphorylation of SNAI1.

### Phosphorylation of SNAI1 by PKD1 occurs in the nucleus and does not alter its localization

**Figure 4:**
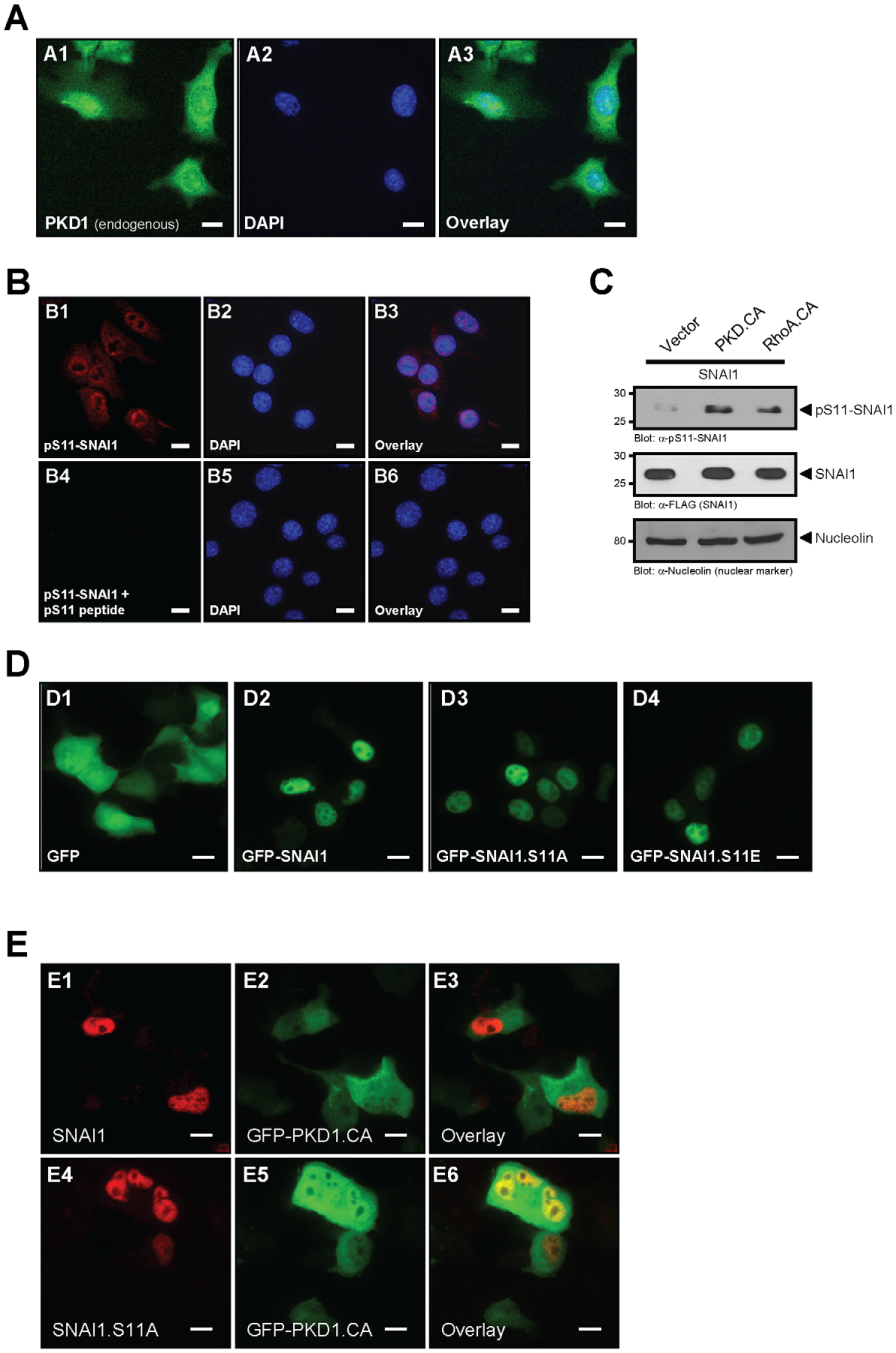
Phosphorylation of SNAI1 by PKD1 occurs in the nucleus and does not alter its localization. **A:** Immunofluorescence staining of NMuMG cells for endogenous PKD1 (anti-PKD1). The bar represents 10 µm. **B:** Immunofluorescence staining of NMuMG cells for S11-phosphorylated SNAI1 (anti-pS11-SNAI1) in absence or presence of competing phospho-S11-peptide and nuclei (DAPI). The bar represents 10 µm. **C:** HeLa cells were transfected as indicated and nuclear extracts were prepared and analyzed by Western blot for SNAI1 (anti-FLAG), pS11-SNAI1 (anti-pS11-SNAI1) and nucleolin (anti-nucleolin, loading control). **D:** NMuMG cells were transfected with GFP control, GFP-SNAI1, GFP-SNAI1.S11A or GFP-SNAI1.S11E mutants. Localization of GFP or GFP-tagged proteins was determined using immunofluorescence analysis (bar is 10 µm). **E:** NMuMG cells were transfected with FLAG-tagged wildtype SNAI1 or SNAI1.S11A mutant and GFP-tagged, active PKD1 (PKD1.CA) as indicated and localization of SNAI1 was determined by indirect immunofluorescence staining (anti-FLAG as primary antibody). The bar represents 10 µm.

Immunofluorescence staining of NMuMG cells with a PKD1-specific antibody and DAPI showed that a subcellular pool of endogenous PKD1 is localized in the nucleus (**Fig. 4A**). Moreover, staining with our anti-pS11 antibody and DAPI showed basal phosphorylation of nuclear SNAI1, most likely due to the basal activity of nuclear PKD1 in these cells (**Fig. 4B**). To confirm specificity, the pS11-SNAI1 antibody was incubated for one hour with a 100-fold molar excess of the pS11-peptide used as antigen prior to use in immunofluorescence. Moreover, immunoblotting of nuclear preparations of control cells and cells with ectopic expression of active PKD1 or RhoA indicated that PKD1 indeed induces phosphorylation of SNAI1 at S11 in the nucleus (**Fig. 4C**). The phosphorylation of SNAI1 by PKD1 did not induce its translocation to the cytosol. This is supported by a mutational analysis, where both, a SNAI1 mutant deficient in the PKD 1 phosphorylation site (SNAI1.S11A), or a SNAI1 mutant with phospho-mimicking mutations for this site (SNAI1.S11E), did not exit the nucleus (**Fig. 4D** and **Fig. S3**). Additionally, the expression of a constitutively-active PKD1 mutant (GFP-PKD1.CA) did not alter the localization of wildtype and S11A-mutated SNAI1 (**Fig. 4E**). Taken together, this indicates that the phosphorylation of SNAI1 at this residue occurs in the nucleus and has no impact on its cellular localization.

### SNAI1 binds to the E-cadherin promoter in presence of active PKD1, but is ineffective in inhibiting E-cadherin expression

**Figure 5:**
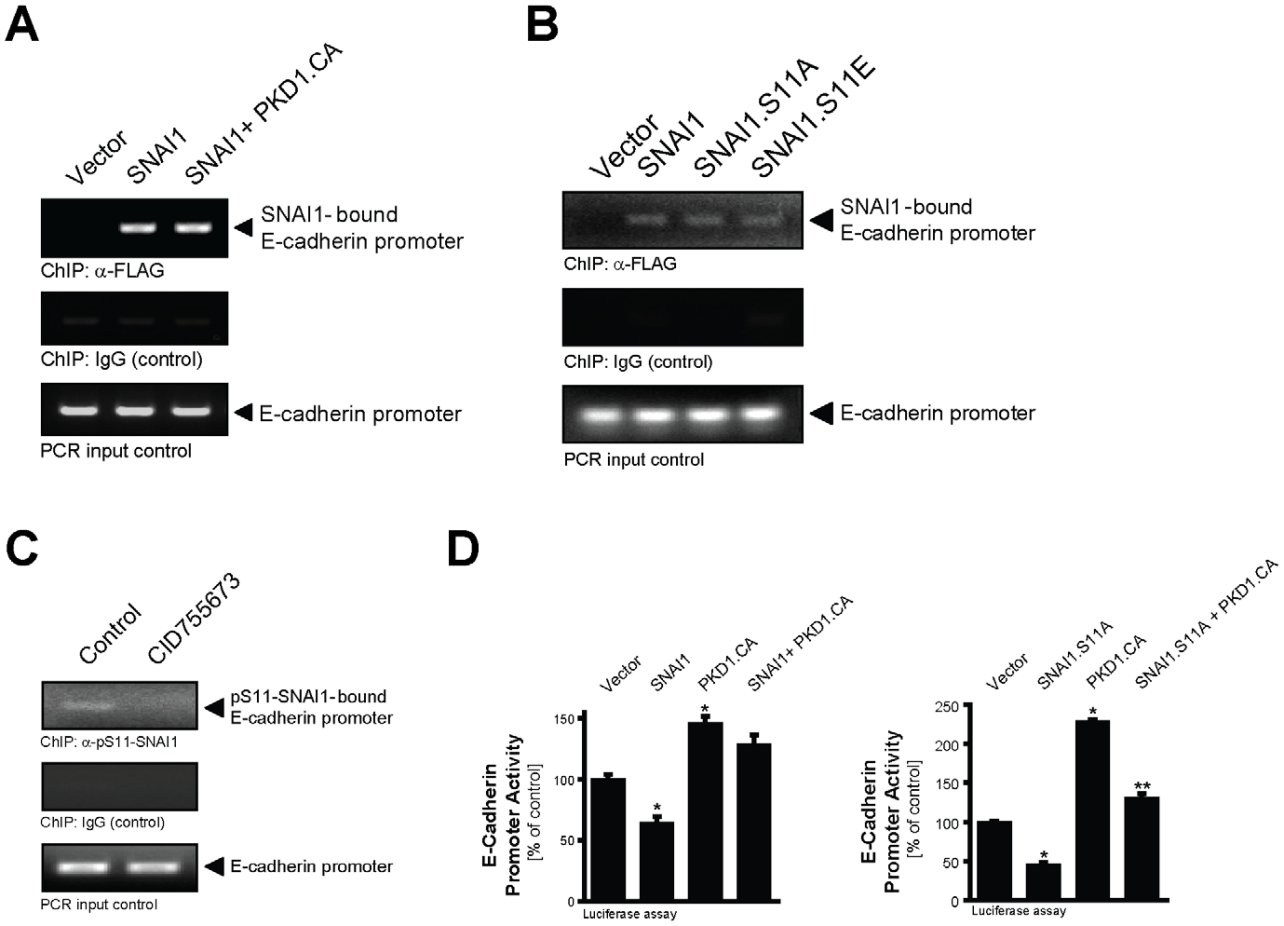
PKD1-regulated SNAI1 binds to the E-cadherin promoter, but is ineffective in its function. **A:** Hek293T cells were transfected with vector control, SNAI1 or active PKD1 (PKD1.CA) and SNAI1 as indicated. SNAI1/DNA complexes were immunoprecipitated (anti-FLAG) after crosslinking and precipitates were analyzed by PCR for the SNAI1-bound E-cadherin promoter. **B:** NMuMG cells were transfected with vector control, SNAI1, SNAI1.S11A or SNAI1.S11E mutants. SNAI1/DNA complexes were immunoprecipitated (anti-FLAG) after crosslinking and precipitates were analyzed by PCR for the SNAI1-bound E-cadherin promoter. **C:** NMuMG cells were treated with CID755673 (25 µM, 1 hr) or left untreated. Phospho-S11-SNAI1/DNA complexes were immunoprecipitated (anti-pS11-SNAI1) after crosslinking and precipitates were analyzed by PCR for the pS11-SNAI1-bound E-cadherin promoter. In experiments depicted in **A–C**, a PCR for the E-cadherin promoter using the input DNA as well as a ChIP using IgG instead of the anti-FLAG antibody served as controls. **D:** Hek293T cells were transfected with vector control, SNAI1 or SNAI1.S11A mutant, active PKD1 (PKD1.CA) or both and E-cadherin promoter luciferase reporter and renilla reporter plasmids. Induced luciferase activity was measured. Error bars shown represent standard deviations. *P* values were acquired with the *t* test, using GraphPad software. Asterisks indicate statistical significance.

Chromatin immunoprecipitation (ChIP) assays showed that in presence of active PKD1 SNAI1 was still bound to the E-Cadherin promoter, suggesting that its phosphorylation by PKD1 does not impact its ability to bind to its target promoter (**Fig. 5A**). This is further supported by the binding of a SNAI1 mutant that mimics phosphorylation (SNAI1.S11E) to the E-cadherin promoter (**Fig. 5B**). Moreover, using the anti-pS11-SNAI1 antibody, S11-phosphorylated SNAI1 was immunoprecipitated with the E-cadherin promoter in NMuMG cells, in which PKD1 is basally active, and inhibition of PKD with CID755673 blocked this interaction (**Fig. 5C** and **Figure S4**). However, the presence of active PKD1 blocked SNAI1-mediated transcriptional repression of E-cadherin expression as measured with E-cadherin gene promoter luciferase assays (**Fig. 5D**, left side). Moreover, a SNAI1.S11A mutant that lacks the phosphorylation site for PKD1 decreased E-cadherin expression (**Fig. 5D**, right side). These data suggest that SNAI1, after its phosphorylation by PKD1, is neither exported from the nucleus, nor does it lose its contact with the E-cadherin promoter, but rather is impacted in its function as a repressor of the E-cadherin gene.

### Phosphorylation of SNAI1 decreases its binding to Ajuba

**Figure 6:**
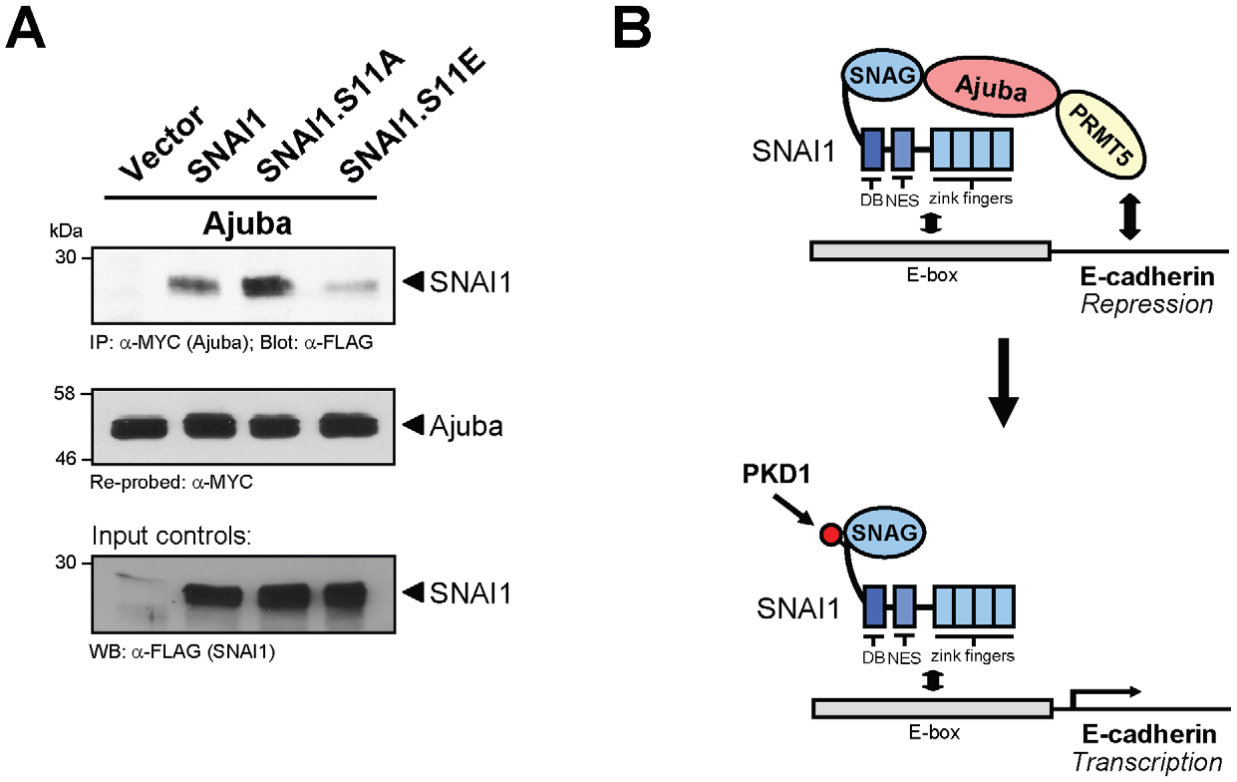
Phosphorylation of SNAI1 decreases its binding to Ajuba. **A:** HeLa cells were co-transfected with MYC-tagged Ajuba and vector control, and FLAG-tagged wildtype SNAI1, SNAI1.S11A or SNAI1.S11E mutants as indicated. Ajuba was immunoprecipitated (anti-MYC) and precipitates were analyzed for co-precipitated SNAI1 (anti-FLAG). Samples were re-stained for Ajuba (anti-MYC) and lysates were control-stained for expressed SNAI1 (anti-FLAG). **B:** Proposed mechanism of how PKD1-mediated phosphorylation regulates SNAI1 function as a transcriptional repressor of E-cadherin gene expression.

Since amino-acid S11 is next to the SNAG domain of SNAI1, which is required for binding to the co-repressor Ajuba, we next analyzed if phosphorylation of this residue impacts the interaction of both proteins. Therefore we determined the interaction of SNAI1 or the S11A or S11E SNAI1 mutants with Ajuba. We found that SNAI1 co-immunoprecipitates with Ajuba and that this interaction is increased when S11 is mutated to alanine, but decreased when mutated to a glutamate mimicking its phosphorylation (**Fig. 6A**). This suggests that PKD1-mediated phosphorylation of SNAI1 at S11 may prevent SNAI1 from binding to Ajuba and from exerting its repressor functions. This mechanism may allow to transiently turn-off SNAI1 functions while it is bound to its target promoter (**Fig. 6B**).

### Loss of nuclear PKD activity and SNAI1 phosphorylation at S11 are markers for invasive breast cancer

**Figure 7:**
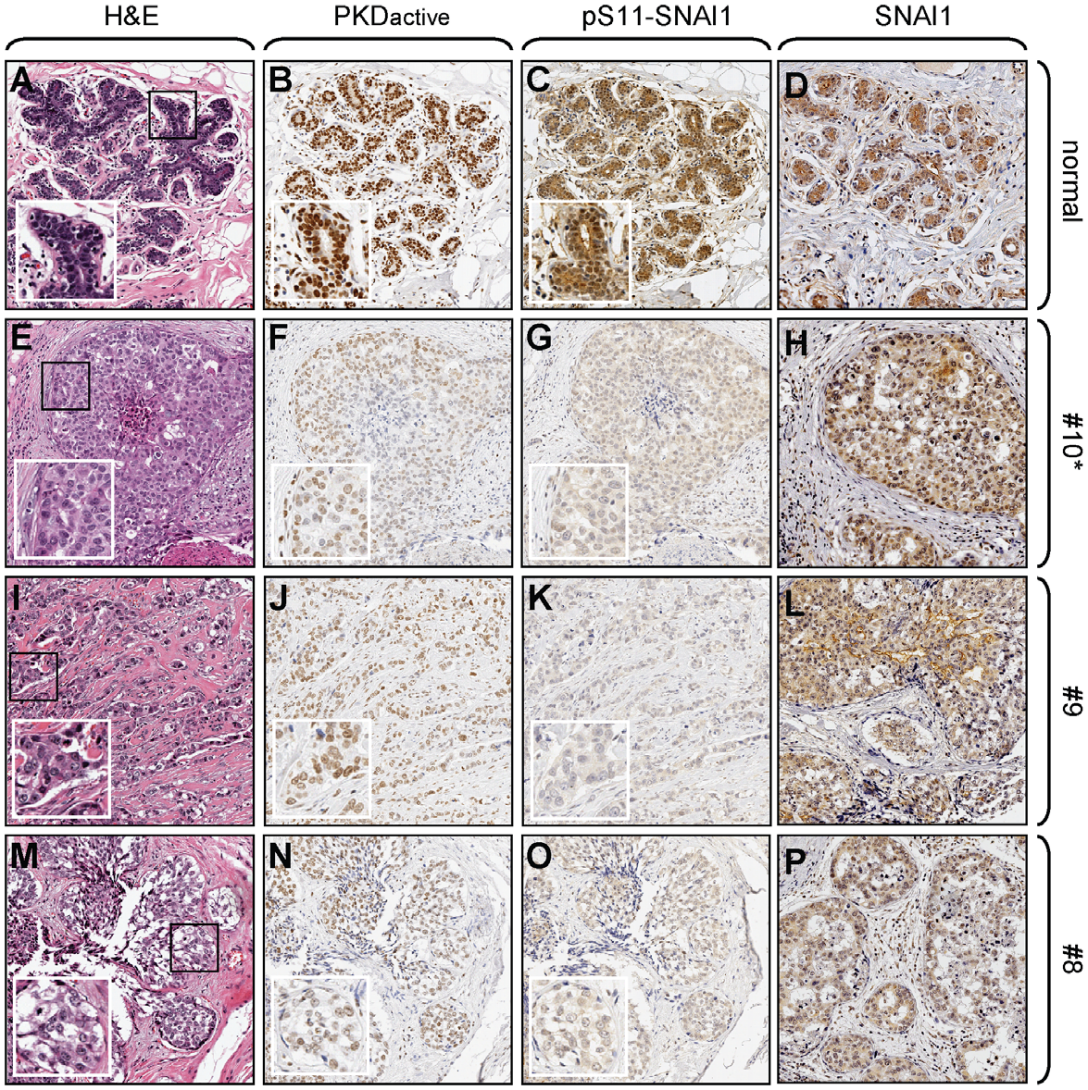
Loss of nuclear PKD activity and SNAI1 phosphorylation at S11 are markers for invasive breast cancer. Tissue microarrays (TMAs) including 10 normal breast tissue samples, 40 invasive ductal carcinoma of the breast and 10 metastatic invasive ductal carcinoma samples from lymph nodes were H&E stained or analyzed for the expression of active PKD (anti-pY95-PKD), S11-phosphorylated SNAI1 (anti-pS11-SNAI1) and total SNAI1 (anti-SNAI1). Representative pictures of normal (**A–D**) and 3 tumor tissues (**E–P**) are depicted. Numbers indicate the position of the tissue on the TMA. The asterisk (sample #10) indicates tumor tissue form a region adjacent to the normal tissue (same patient). Inserts show enhanced area.

To determine *in vivo* relevance of our data obtained with cell culture, we utilized our phosphospecific antibody directed against PKD-phosphorylated SNAI1 in tissue microarrays of human normal breast tissue and invasive ductal carcinoma (IDC). 40 tumor samples as well as 10 normal samples were analyzed. A representative selection of samples is presented in **Fig. 7**. We found that nuclear localization of active PKD1 in normal ductal epithelia of the breast correlates with SNAI1 phosphorylation at S11 in the nuclei of these cells (**Figs. 7B** and **7C**). Moreover, in all samples of IDC with decreased PKD activity a correlating decrease in SNAI1 phosphorylation at S11 in the nucleus of tumor cells was detected (**Fig. 7E–P**), whereas total SNAI1 levels were comparable in all samples including normal ductal epithelium. To confirm specificity, the pS11-SNAI1 antibody was incubated for one hour with a 100-fold molar excess of the pS11-peptide used as antigen prior to use in immunohistochemistry (**Fig. S5**).

## Discussion

Epithelial-mesenchymal transition (EMT), a program that is activated in development, but also in adult tissue under pathological conditions including fibrosis and epithelial neoplasia, allows epithelial cells to acquire the phenotypical characteristics and behavior of mesenchymal cells [46], [47]. Normal mammary gland epithelial cells NMuMG are a *bona fide* system for TGFβ1-induced EMT [48]. Here we show that TGFβ1-induced downregulation of PKD1 activity is a significant factor contributing to EMT in these cells (**Fig. 1**). This is underlined by constitutive expression of a kinase-dead allele of PKD1, which leads to a similar mesenchymal phenotype as observed after long-term treatment with TGFβ1 (**Fig. 1**).

In prostate epithelia cells, PKD1 has been shown to stabilize cell adhesions through interaction with E-cadherin and phosphorylation of β-catenin [43], but also by regulation of E-cadherin expression [27]. Our data suggests that presence of active PKD1 maintains the epithelial phenotype of normal breast epithelial cells. One of the mechanisms of how PKD1 may achieve this is by maintaining high E-cadherin expression levels. Indeed a kinase-dead PKD1 decreased E-cadherin expression levels (**Fig. 2**). Decreased expression of E-cadherin is a pivotal event in EMT, and in tumor cells it occurs through epigenetic silencing as well as through zinc finger transcriptional repressors such as SNAI1 [12]. Downregulation of E-cadherin was described for many epithelial tumor types and is linked to poor prognosis and increased invasion and metastasis [2]. Interestingly, induced expression of E-cadherin can cause a reversion of the mesenchymal to an epithelial phenotype (mesenchymal-epithelial transition) [11]. Based on our results it will be interesting to test in future studies if a re-expression of PKD1 in mesenchymal cell types can induce such a transition to an epithelia phenotype.

In human samples of invasive ductal carcinoma (IDC), PKD1 is downregulated in its expression [37] and activity (**Fig. 7**). Additionally, PKD1 is downregulated in invasive breast cell lines and a re-expression of active PKD1 completely blocks their invasiveness in 2D and 3D cell culture [37]. Moreover, the depletion of PKD1 from non-invasive MCF-7 using reverse genetics induced invasiveness [37]. Similar functions for PKD1 on tumor cell invasion were described for other cancers including prostate and gastric cancer [38], [39]. This suggests that in breast cancer PKD1 is a molecular switch that regulates motility and its effects on E-cadherin expression and EMT may be one of the mechanisms it uses.

Our data suggest that PKD1 regulates E-cadherin expression through phosphorylation of SNAI1. Phosphorylation of SNAI1 at S11 was described by several laboratories [27], [28] and initially PKA was identified as the kinase mediating this phosphorylation *in vitro* [28]. Recently, it was shown that PKD1 also can contribute to SNAI1 phosphorylation at S11 [27]. Utilizing purified PKD1 and bacterially-expressed SNAI1 as well as a newly-generated phosphospecific pS11-SNAI1 antibody, we now provide proof that SNAI1 is a direct target for PKD1 (**Fig. 3**). We also show that this phosphorylation occurs in cells in response to ectopic PKD1 expression and to PKD1 activation (**Fig. 3**).

Phosphorylation of SNAI1 mediates its binding to 14-3-3 proteins [27], [29], however, the consequence of this interaction can have different outcomes. It was suggested that the formation of complexes with 14-3-3 stabilizes SNAI1 interaction with its co-repressors [29]. Others have shown a 14-3-3σ-mediated nuclear export and inactivation of SNAI1 after its phosphorylation at S11 [27]. SNAI1 exclusion from the nucleus and proteosomal degradation is mediated through its phosphorylation by GSK3β [18] and at this point it is unclear if a connection between both phosphorylation pathways exists. In NMuMG cells neither mimicking of the phosphorylation, nor phosphorylation of SNAI1 at S11 by active PKD1 led to its nuclear exclusion (**Fig. 4**). Instead, after phosphorylation at S11, we observed a decrease in the interaction of SNAI1 with its co-repressor Ajuba (**Fig. 6**). Since S11 is located next to the SNAI1 SNAG domain, which contributes to Ajuba-SNAI1 complex formation [22], we propose that phosphorylation of this residue may hinder this interaction. This would explain why PKD1 increases E-cadherin promoter activity although SNAI1 is still promoter-bound (**Fig. 5**). Such a mechanism is a novel concept for SNAI1 regulation. It would allow transient quick changes in gene regulation of epithelial cells, without inducing SNAI1 degradation. It is possible that this mechanism is also utilized when cancer cells switch to an invasive tumor type or when metastases are established at distant sites.

*In vivo* studies support these results. PKD1 activity and SNAI1 phosphorylation at S11 occur in normal breast ductal epithelium and are decreased in IDC. This data not only confirms data obtained in cell culture, but also indicates that SNAI1 is expressed in normal ductal cells of the breast, where it is silenced by phosphorylation (**Fig. 7**). However, this correlation needs to be addressed in more detail in future studies with increased numbers of tumor and normal samples to obtain statistical power. At this point it is unclear why normal breast cells (**Fig. 4A**) and normal ductal tissue (**Fig. 7A–D**) express SNAI1, but keep it in an S11-phosphorylated, inactive state. One explanation is that some aspects of nuclear SNAI1 signaling may be needed for maintaining normal tissue or cell functions. Another possibility is that cells utilize this mechanism to facilitate a rapid switch of gene expression. Such signaling may be important for breast development and involution, or when tumor cells switch from a motile state to a more epithelial phenotype.

Taken together, our data show that PKD1 maintains the normal epithelial phenotype by preventing EMT. As one possible mechanism, we identify the direct phosphorylation of SNAI1, an event that occurs in the nucleus and transiently turns off SNAI1 inhibitory functions, while it stays bound to its target E-cadherin promoter. Our results contribute to the understanding of the complex molecular mechanisms which regulate the transition from normal epithelial cells to invasive tumor cells and may lead to new insights into how this process may be inhibited or reversed.

## Materials and Methods

### Cell Lines, Antibodies and Reagents

MCF-7, MDCK, Hek293T, and Panc1 cells were maintained in DMEM with 10% FBS. All cell lines were obtained from American Type Culture Collection ATCC (Manassas, VA). NMuMG cells were cultivated in DMEM plus 10% FBS and 10 µg/ml insulin. TGFβ1 was from Peprotech (Rocky Hill, NJ). Anti-GST, anti-PKD1 and anti-14-3-3 antibodies were from Santa Cruz (Santa Cruz, CA), anti-HA, anti-FLAG (M2), anti-actin, anti-nucleolin from Sigma-Aldrich (St Louis, MO), anti-E-cadherin from BD Biosciences (San Diego, CA), anti-N-cadherin from Epitomics (Burlingame, CA), anti-Snail (ab85931), anti-cytokeratin, anti-MYC and anti-GFP from Abcam (Cambridge, MA), anti-pMOTIF (PKD substrate antibody) and anti-pS744/742-PKD antibody (recognizes S738/S742-phosphorylated PKD1) from Cell Signaling Technology (Danvers, MA). A rabbit polyclonal antibody specific for human SNAI1 phosphorylated at S11 (anti-pS11-SNAI1 antibody) was raised by 21 Century Biochemicals (Marlboro, MA) using Ac-FLVRKP[pS]DPNRKPC-amide and Ac-CFLVRKP[pS]DPNRKPN-amide peptides as antigens. Secondary HRP-linked antibodies were from Roche (Indianapolis, IN). Secondary antibodies Alexa Fluor 568 F(ab′)2 fragment of goat-anti-mouse IgG or Alexa Fluor 546 F(ab′)2 fragment of goat-anti-rabbit were from Invitrogen (Carlsbad,CA). Mirus HeLa-Monster (Mirus, Madison, WI) and Superfect (Qiagen, Valencia, CA) were used for transient transfection of HeLa, Mirus TransIT-293 (Mirus) for Hek293, Lipofectamine 2000 (Invitrogen) for Panc1 and MDCK, and GenJet (SignaGen Laboratories, Ijamsville, MD) for NMuMG. Bradykinin and 12-phorbol 13-myristate acetate (PMA) were from Sigma, EGF (epidermal growth factor) from PeproTech (Rocky Hill, NJ) and CID755673 (2,3,4,5-Tetrahydro-7-hydroxy-1*H*-benzofuro[2,3-*c*]azepin-1-one) from Tocris Bioscience (Ellisville, MO). The PKD-specific substrate peptide used was AALVRQMSVAFFFK.

### DNA Constructs

GFP-tagged human SNAI1 was obtained from Addgene (Cambridge, MA) [18]. The expression construct for FLAG-tagged SNAI1 was generated using above construct as a template and 5′-GCGGGATCCATGCCGCGCTCTTTCCTCGTCAGG-3′ and 5′-CGCCTCGAGTCATTTGTCATCATCGTCCTTATAGTCGCGGGGACATCCTGAGCAGCCGGA-3′ as primers and by cloning into pcDNA4/TO (Invitrogen, Carlsbad, CA) via BamHI and XhoI. The expression construct for a GST-SNAI1 fusion protein was generated by cloning the same fragment into pGEX4-T1 via BamHI and XhoI. Mutagenesis was carried out using the QuikChange kit (Stratagene, La Jolla, CA). The SNAI1.S11A mutants were generated using 5′-CTCGTCAGGAAGCCCGCCGACCCCAATCGGAAG-3′ and 5′-CTTCCGATTGGGGTCGGCGGGCTTCCTGACGAG-3′, and the SNAI1.S11E mutants using 5′-CTCGTCAGGAAGCCCGAGGACCCCAATCGGAAG-3′ and 5′-CTTCCGATTGGGGTCCTCGGGCTTCCTGACGAG-3′ as primers. The expression construct for MYC-tagged Ajuba was generated by amplification of human Ajuba from a HeLa cDNA library using 5′-GCGCTCGAGTCAGATATAGTTGGCAGGGGGTTG-3′ and 5′-GCGGGATCCATGGAACAAAAACTCATCTCAGAAGAGGATCTGGAGCGGTTAGGAGAGAAAGCCAGT-3′ as primers and by cloning into pcDNA4/TO via BamHI and XhoI. Expression plasmids for HA-tagged or GFP-tagged wildtype, constitutively-active or kinase-inactive PKD1 were described before [41]. The luciferase reporter construct pGL3-E-cadherin promoter (−178/+92) from A. Garcia de Herreros, and the expression construct for constitutively-active RhoA was described previously [41]. The use of shRNA specifically-directed against human PKD1/2 was described elsewhere [37], [41]. The lentiviral shRNA expression system to knock-down mouse PKD1 and PKD2 expression is commercially available from Sigma (SHDNA MISSION® shRNA Plasmid DNA; St. Louis, MO, USA). Sequences used were NM_008858.1-2104s1c1 and NM_178900.2-827s1c1. The ViraPower Lentiviral Expression System (Invitrogen) was used for an optimized mix of packaging plasmids to produce Lentivirus in 293FT cells.

### Cellular extracts, Immunoblotting, Immunoprecipitation and PAGE

#### Cellular lysates

Cells were washed twice with ice-cold PBS (140 mM NaCl, 2.7 mM KCl, 8 mM Na_2_HPO_4_, 1.5 mM KH_2_PO_4_, pH 7.2) and lysed with RIPA buffer (0.01 M NaHPO_4_ pH 7.2, 2 mM EDTA, 50 mM NaF, 150 mM NaCl, 0.1% SDS, 1% sodium deoxycholate, 1% Nonindet P-40) for total cell lysates, or Buffer A (50 mM Tris-HCl pH7.4, 1% Triton X-100, 150 mM NaCl, 5 mM EDTA pH 7.4) plus Protease Inhibitor Cocktail (PIC, Sigma-Aldrich, St. Louis, MO). Lysates were vortexed and incubated on ice for 30 min. Following centrifugation (13,000 rpm, 15 min, 4°C) the supernatant was collected and subjected to SDS-PAGE (Western blotting) or proteins of interest were immunoprecipitated by 1 hr incubation with a specific antibody (2 µg) followed by 30 min incubation with protein G-Sepharose (Amersham Biosciences). Immune-complexes were washed 3 times with TBS (50 mM Tris-HCl pH 7.4, 150 mM NaCl), resolved in 20 µl TBS and 2× Laemmli buffer and subjected to SDS-PAGE.

#### Nuclear extracts

Cells were washed twice with ice-cold PBS, scraped in NE buffer I (10 mM HEPES pH 7.9, 10 mM KCl, 0.1 mM EDTA, 0.1 mM EGTA, 1 mM DTT, 1 mM PMSF), incubated on ice (15 min) and supplemented with 10% NP-40. Samples were lysed on a shaker (4°C, 2 min), centrifuged (RT, 1 min, 13,000 rpm) and supernatant (cytosolic lysate) and pellets (nuclei) were collected. Nuclei were lysed in NE buffer II (20 mM HEPES pH 7.9, 0.4 M NaCl, 1 mM EDTA, 1 mM EGTA, 1 mM DTT, 1 mM PMSF) by rough shaking for 20 min at 4°C. Samples were then centrifuged (5 min, 4°C, 15,000 rpm) to obtain nuclear lysates. Following separation by SDS-PAGE, samples were transferred to nitrocellulose membranes and visualized by immunostaining.

### Reporter Gene Assays

Cells were transiently transfected with 2 µg pGL3-E-cadherin promoter (-178/+92) luciferase reporter construct, 0.1 µg renilla luciferase reporter and 1 µg of the cDNA of interest. 24 hr after transfection cell lysates were prepared by washing cells twice with ice-cold PBS, scraping in 250 µl Passive Lysis Buffer (Promega) and centrifugation (13,000 rpm, 10 min, 4°C). Assays for luciferase activity were performed on total cell lysates using a Veritas luminometer (Symantec, Cupertino, CA). Protein expression was controlled by immunoblot analysis.

### Chromatin Immunoprecipitation (ChIP) Assay

ChIP assays were performed using the EZ-ChIP™ Chromatin Immunoprecipitation (ChIP) KIT from Millipore (Bedford, MA) according to the manufacturer's protocol. 4 µg primary antibody (anti-FLAG, anti-pS11-SNAI1) or IgG control were used for immunoprecipitations. Immunoprecipitates were analyzed by PCR using the following primer set: 5′-AATCAGAACCGTGCAGGTCC-3′ and 5′-ACAGGTGCTTTGCAGTTCCG-3′ to amplify a 250 bp fragment of the human E-cadherin promoter corresponding to an E2-box binding site or a previously-described primer set to detect a 360 bp fragment of the mouse E-cadherin promoter [41].

### *In Vitro* Kinase Assays

Kinase assays with GST fusion proteins were carried out by adding 250 ng of active, purified PKD1 (Upstate, Charlottesville, VA) to 2 µg of purified GST-fusion protein in a volume of 40 µl kinase buffer (50 mM Tris pH 7.4, 10 mM MgCl_2_ and 2 mM DTT) supplemented with 100 µM ATP. The kinase reaction (30 min, RT) was stopped by adding 2× Laemmli buffer. Kinase assays to determine the activity of immunoprecipitated PKD1 were carried out as follows: PKD was immunoprecipitated and 20 µl kinase buffer (50 mM Tris/HCI pH 7.4, 10 mM MgCl_2_, 2 mM dithiothreitol) was added to the precipitates. The kinase reaction was started by addition of 10 µl of kinase substrate mixture (150 µM PKD-specific substrate peptide, 50 µM ATP, 10 µCi of [γ-^32^P]ATP in kinase buffer) and carried out for 30 min at 37°C. The reaction was stopped and samples were spotted onto P81 phosphocellulose filters (Whatman). Samples were washed 3 times with 0.75% phosphoric acid, once with acetone, air dried and radiolabel incorporation was determined with scintillation counting. To determine stoichiometry, purified SNAI1 (3 µg) was incubated with active PKD1 (100 ng) and 100 µM [γ-^32^P]ATP in kinase buffer for 60 min at 37°C. The reaction was stopped and aliquots were spotted onto P81 phosphocellulose filters. Samples were further processed as above.

### Immunofluorescence Microscopy

Cells were transfected or treated as indicated in 8 well ibiTreat μ-Slides (Ibidi, Martinsried, Germany). The next day cells were washed twice with phosphate-buffered saline (PBS), fixed with 4% paraformaldehyde (15 min, 37°C), washed three times in PBS, permeabilized with 0.1% Triton X-100 in PBS for 2 min at room temperature (RT) and then blocked with 3% bovine serum albumin and 0.05% Tween 20 in PBS (blocking solution) for 30 min at RT. Samples were incubated for 2 hours at RT with primary antibodies (anti-E-cadherin 1∶500, anti-pS11-SNAI1 1∶5000, or anti-FLAG 1∶4000 for overexpressed SNAI1 and SNAI1 mutants) diluted in blocking solution. Cells were washed five times with PBS and incubated with secondary antibodies (Alexa Fluor 568 F(ab′)2 fragment of goat-anti-mouse IgG or Alexa Fluor 546 F(ab′)2 fragment of goat-anti-rabbit; both Invitrogen), diluted (1∶500) in blocking solution for 2 hours at RT. Nuclei were stained with DAPI. After extensive washes in PBS, cells were mounted in Ibidi mounting medium (Ibidi). Samples were examined using an IX81 DSU Spinning Disc Confocal from Olympus with a 40× objective. Images were processed using NIH ImageJ.

### Tissue Microarrays

Tissue array slides containing histologically-confirmed human breast cancer and normal human breast tissue samples (IMH-364/CBA2) were purchased from Imgenex (San Diego, CA). The TMAs were deparaffinized (one hour at 60°C), dewaxed in xylene (five times for four minutes) and gradually rehydrated with ethanol (100%, 95%, 75%, twice with each concentration for three minutes). The rehydrated TMAs were rinsed in water and subjected to antigen retrieval in citrate buffer (pH 6.0) as described by the manufacturer (DAKO, Carpinteria, CA, USA). Slides were treated with 3% hydrogen peroxide (five minutes) to reduce endogenous peroxidase activity and washed with PBS containing 0.5% Tween 20. Active PKD1 and S11-phosphorylated SNAI1 were detected using specific antibodies (anti-pY95-PKD at 1∶200 and anti-pS11-SNAI1 at 1∶1000) in PBS/Tween and visualized using the Envision Plus Dual Labeled Polymer Kit following the manufacturer's instructions (DAKO, Carpinteria, CA, USA). H&E staining was performed as previously described [37]. Images were captured using ImagePro software (Media Cybernetics, Bethesda, MD, USA).

### Statistical Analysis

Data are presented as mean ± SD. P values were acquired with the student's *t*-test using Graph Pad software, and p<0.05 is considered statistically significant.

## Supporting Information

Figure S1 **PKD1 regulates E-cadherin expression in MDCK cells.** MDCK cells were transfected with vector control, constitutively-active PKD1 (PKD1.CA) or kinase-dead PKD1 (PKD1.KD). After 72 hours, samples were analyzed by Western blot for E-cadherin expression (anti-E-cadherin), as well as ectopic expression of PKD1 mutants (anti-PKD1). Staining for actin (anti-actin) served as loading control. This data shows that PKD1 regulates E-cadherin expression in MDCK cancer cells. It also shows that the kinase-dead PKD1 can act as dominant-negative mutant.
(EPS)

Figure S2 **Control blots for** **Fig. 3E and 3F** **.** Lysates of the samples were control-stained for GST-RhoA.CA or GST expression using anti-GST antibody.
(EPS)

Figure S3 **Blunting or mimicking mutations for pS11 do not impact SNAI1 localization.** Panc1 cells were transfected with GFP control, GFP-SNAI1, GFP-SNAI1.S11A or GFP-SNAI1.S11E mutants. Localization of GFP or GFP-tagged proteins was determined after 16 hrs using immunofluorescence analysis (bar is 25 µm).
(EPS)

Figure S4 **The anti-pS11-SNAI1 antibody specifically immunoprecipitates PKD1-phosphorylated SNAI1.** Hek293T cells were transfected with vector control, FLAG-SNAI1 or active PKD1 (PKD1.CA, PKD1.S738E.S742E) as indicated. Phospho-S11-SNAI1 was immunoprecipitated (anti-pS11-SNAI1) and samples were subjected to SDS-PAGE and analyzed in immunoblots using an anti-FLAG antibody to detect immunoprecipitated SNAI1. Control Western blots were performed to detect PKD1.CA input (anti-PKD1) or for vimentin (anti-vimentin) as loading control.
(EPS)

Figure S5 **Specificity of nuclear pS11-SNAI1 staining in normal ductal breast tissue.** Normal breast tissue was immunohistochemically-stained with anti-pS11-SNAI1 (left side). To confirm specificity, the anti-pS11-SNAI1 antibody was incubated for one hour with a 100-fold molar excess of the pS11-peptide used as antigen prior to use in immunohistochemistry (right side). This data shows that obtained results showing pS11-SNAI1 in nuclei of normal breast ductal tissue represent specific staining.
(EPS)
